# Assessment of Flexible Pavement Containing Rubberized Asphalt

**DOI:** 10.3390/polym18080927

**Published:** 2026-04-10

**Authors:** Noorance Al-Mukaram, Tariq Al-Mansoori, Ali M. Lafta, Karzan Ismael, Pooyan Ayar

**Affiliations:** 1 Department of Civil Engineering, College of Engineering, Al-Muthanna University, Samawah 66001, Iraq; tariq.almansoori@mu.edu.iq (T.A.-M.); ali.majd@mu.edu.iq (A.M.L.); 2 Department of City Planning Engineering, Technical College of Engineering, Sulaimani Polytechnic University, Sulaymaniyah 46001, Iraq; 3 School of Civil Engineering, Iran University of Science and Technology (IUST), Tehran 16846-13114, Iran; ayar@iust.ac.ir

**Keywords:** Marshall tests, modified asphalt, waste tires, rubberized asphalt, rutting test

## Abstract

This work deals with a practical method of using crumb rubber resulting from waste tires to produce modified bitumen via a wet mixing method for road construction in Iraq. Due to wide variation in temperatures and over-loading traffic in Iraq, rutting deformation is the most observed structural pavement problem. Also, tire wear and tear are higher in Iraq than in other countries due to high temperature and dry weather most of the year, which makes considerable amounts of waste tire piles easily accessible. Utilizing this waste material could be crucial to the environment and economy of the country, as well as to the sustainability of resources. Using waste tire materials as bitumen modifiers in the production of hot mix asphalt is a widely practiced experiment, although it is applied differently depending on the weather, type of bitumen used, and its availability. In the methodology of this research, it is suggested to modify asphalt grades 60/70 by a certain amount of crumb rubber (5–20%). The modified asphalt and asphalt grade 40/50 were used in preparing two types of asphalt concretes to examine their volumetric properties and evaluate their rutting behavior. The results for both mixtures were compared to the Iraqi General Specifications for Roads and Bridges (SORB/R9). The findings showed significant improvements in Marshall stability and flow, as well as in the percentages of voids satisfied in the modified mixture. After using rubberized asphalt in the mixture, the rutting depth was recorded below 20 mm and decreased by 30% and 26% at temperatures of 40 °C and 60 °C, respectively, compared to the controlled mixture.

## 1. Introduction

Applying asphalt concrete to pave roads is a widely known technique because of its cost-effectiveness, its long-term life for road construction, and because it is easy to install and maintain under prevalent traffic and weather conditions. Different techniques have been utilized to enhance road surface conditions and management, such as geographical information systems (GIS) technology [1,2]. However, flexible pavement usually undergoes heavy traffic loads and extreme climates that affect pavement quality and result in different surface deformations, such as rutting, bleeding, patches, and cracks [3,4,5].

Because of the growing attention to environmental sustainability, there is a need to discover innovative solutions for developing transport infrastructure. The aim is the adoption of highly sustainable practices (i.e., reducing the use of materials derived from fossil natural resources, thus limiting climate change-related issues). Numerous conducted studies have referred to the fact that adding different materials or additives to asphalt pavement can significantly enhance pavement performance and promote sustainable engineering. The recycling aspect is widely applied in the mix design. For example, using recycled concrete aggregate and plastic waste in the design of concrete mixture and hot mix asphalt [6,7,8,9]. Moreover, the use of crumb rubber (CR) is a technique that has been in use since the 1960s and which has received increasing attention and success in the field of pavement engineering throughout the world since the early 2000s. This sustainable aspect not only addresses the environmental concerns associated with tire disposal but also aims to improve the physical and chemical properties of asphalt binders and mixtures. The addition of a specific amount of recycled tire rubber as a replacement for conventional pavement materials might achieve promising results. Despite the fact that the cost of this sustainable practice remains high, production of CR is an enhancement for extending service life and reducing maintenance works that are needed in rubberized pavements compared to non-rubberized pavements [10]. According to Labbafi et al. [11], the rubberized mixture showed 25% improvement in performance compared with the conventional mixture, since using ground waste tires in the flexible pavement may enhance its performance and durability, as well as improve crack and temperature resistance. The contribution of CR in asphalt concrete is described as ‘dry’ and ‘wet’ mixing processes. In the ‘dry’ method, CR is introduced as a partial replacement of fine aggregate. In contrast, the ‘wet’ method includes adding CR to asphalt cement to enhance its rheological and physical properties. Subsequently, rubber particles have non-uniform shapes as well as a large surface area, which makes them react and blend efficiently with asphalt at high temperatures in the wet process [12].

According to previous studies [13,14,15,16], adding CR in a percentage between 10 and 20% by weight to an asphalt binder using the method of wet mixing provides better overall performance based on Marshall stability, thermal cracking, creep resistance, and water sensitivity. Also, Bahia and Davies [17] applied an amount of CR that equaled 15% of the total weight of the rubber–asphalt mixture, and the modified asphalt showed a significant enhancement in the elasticity and deformation resistance. Moreover, several studies [18,19,20,21,22] have shown that the use of crumb rubber in asphalt mixtures can enhance pavement durability and reduce maintenance frequency, which indirectly contributes to lowering energy consumption and CO_2_ emissions associated with road construction and rehabilitation. This supports the broader role of sustainable transport infrastructure materials in promoting green development and environmental. Further optimization can be applied to rubber particles by using either microwave treatment or chemical modifications to enhance mechanical characteristics such as ductility, elasticity, and toughness. The reported findings of modified asphalt concrete highlighted better resistance to rutting and fatigue by 2.5 and 10 times, respectively [23].

Furthermore, the aging resistance of rubberized binders was significantly enhanced, thereby extending pavement life can be achieved. According to Sarsam and Al-Sadik [24], stiffer asphalt was obtained after adding 8% CR to asphalt grade 40/50, which might result in reducing penetration and ductility by 47.24% and 49.6%, respectively. As well, average increases of 10.75% and 79.25% were confirmed in the softening point and creep stiffness, respectively. Finally, rutting resistance after 1000 repetitions was enhanced by 57.3% and 32.4 for long- and short-term aging, respectively. On the other hand, a study was conducted by Joni and Abed [25] to investigate the effect of CR percentage on the modified asphalt concrete. They indicated that adding 7.5% of CR to modify the asphalt grade 40/50 resulted in improvements of the retained strength index and indirect tensile strength ratio by 13% and 9%, respectively. Also, other researchers [26] revealed that the rutting depth in the asphalt mixture decreased by 57.5% after modifying the asphalt grade 40/50 with 10% CR compared to the control mixture.

In this research, two roads under construction in Samawah city were selected as case studies. The properties of the mix design were investigated, and several experimental works were suggested. The reuse of rubber shingles not only improves the mechanical properties of the mixture but also provides new ideas in waste recycling and reuse. The novelty of this study lies in its focus on wet modification of a special type of asphalt in Iraq (Asphalt 60/70) that has not been comprehensively addressed in the existing works. Crumb rubber was used in the wet modification process of asphalt 60/70 to produce rubberized asphalt for enhancing the performance of asphalt concrete. The rubberized asphalt will be used as a replacement for asphalt 40/50 in the reference mixture. As a result, these modified surface layer mixtures may increase pavement stiffness by resisting traffic loads and variation in temperatures, and thereby reduce pavement deformation.

The remaining sections are organized as follows: Section 2 presents the materials and methods used in this study. Section 3 describes the experimental work. Section 4 provides the results and a discussion. Section 5 outlines the conclusions and recommendations.

## 2. Materials and Methods

**Figure 1:**
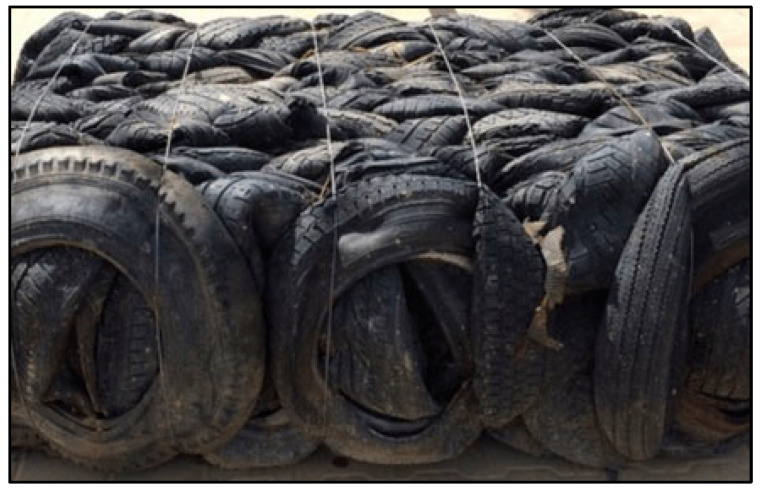
Scrap tires used in the current work.

**Figure 2:**
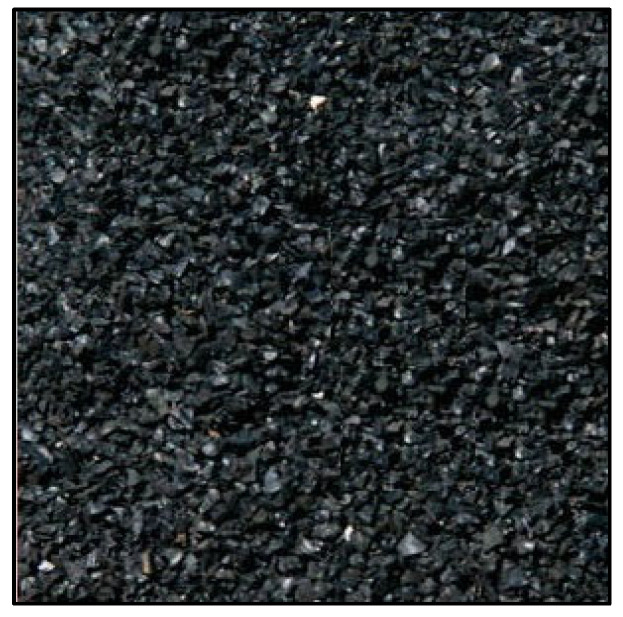
Size of CR particles after sieving in the current work.

This section describes the materials used for the experimental work. Building materials were locally produced in Iraq and used for modifying asphalt and preparing asphalt concrete, as described in the following:

- Asphalt: Two types of asphalt were used in the current work, bitumen of grades 40/50 and 60/70. Both types are produced in the Samawah oil refinery in Iraq for use in road pavement. Bitumen of grade 40/50 will be used for preparing the control mix, whereas bitumen of grade 60/70 will be modified with crumb rubber to represent the modified asphalt mix. The properties of the two types of asphalt can be shown in Table 1:

- Natural aggregates: These materials were selected from the Najaf quarry. Aggregates were graded into different sizes by sieve analysis. Table 2 and Table 3 demonstrate the physical properties of natural fine and coarse aggregates, respectively.
- Filler material: Limestone dust was produced in the Karbala quarry and used as mineral filler in the current work. The physical properties can be shown in Table 4.
- Waste tires: In the current study, scrap tires were collected from the Diwaniya rubber factory, as shown in Figure 1. Generally, tires consist of several materials, including 48% rubber hydrocarbon, 22% silica and carbon black, 15% reinforcing belts, 5% fabric cords, and 10% mix of oil, stearic acid, wax, ZnO, antidegradants, etc. [27]. The metal reinforcements of tires should be removed, and then a process of washing, cutting, and shredding is applied to the material, as depicted in Figure 2. For the purposes of this study, the CR particles were sieved, and the ones that passed through sieve No.8 (2.36 mm opening size) and were retained on sieve No.50 (0.3 mm size) were used.

## 3. Experimental Works

Several laboratory works were conducted in this research following the standard tests. Firstly, the wet method [28] was applied to produce crumb rubber asphalt by adding certain amounts of waste tires. Secondly, production of asphalt concrete (AC) was performed on two parts: a reference mixture using asphalt grade 40/50 and a modified mixture with crumb rubber asphalt. Following that, a number of volumetric characteristics (i.e., Marshall tests [29]) and the Hamburg wheel track test [30] were conducted to obtain an estimation of the rutting resistance of rubberized pavement at temperatures of 40 °C and 60 °C, which are very close to the temperatures of pavement during the summer season in Iraq.

### 3.1. Preparation of Rubberized Asphalt

As presented in previous studies [16,22,31], different polymers can be added to modify bitumen performance by applying a wet mixing process and to achieve sustainability. Crumb rubber (CR) was selected as a bitumen modifier after the sieving process. Then, bitumen 60/70 grade was modified by adding specific contents of CR in the range (5–20%) by weight of bitumen. To obtain homogeneous or consistent asphalt, the mixing process was performed at a temperature of 170 °C for a 20 min period using a propeller mixer at a speed of 700 rpm. The outcomes of adding 20% CR to asphalt 60/70 showed that the measured penetration and ductility were decreased by 33% and 37%, respectively. In addition, the softening point was improved to reach 56 °C after adding 20% of CR. As the CR was added to hot bitumen, the content of aromatic oil in the bitumen decreased by diffusing, causing an increase in the rubber volume. Moreover, the decrease in oil content resulting from the digestion process might enhance the viscosity of bitumen and could form a thick layer that covers aggregates in the hot mix asphalt (HMA) mixture. Subsequently, increasing the thickness of this layer results in more durable pavement and improves its resistance to aging, rutting, and fatigue cracks [32,33,34].

It is worth mentioning that the characteristics of modified bitumen have a similarity with those of bitumen grade 40/50 (see Table 1) after adding CR amount between 15% and 20%. The physical properties of the rubberized asphalt can be presented in Table 5 following the standard tests. These findings also complied with the SORB/R9 [35] requirements. However, adding more than 20% of CR necessitates an increase in mixing time and temperature. This might cause polymer swelling due to the diffusion of solvent molecules, and then a decrease in the molecular weight of asphalt will be obtained [36].

### 3.2. Preparation of Asphalt Concrete

Following the ASTM D6926 procedures, two types of asphalt concrete (AC) were prepared for testing in the lab: the first was conventional asphalt concrete, including 40/50 asphalt, whereas the second mixture was designed as a sustainable mixture containing crumb rubber asphalt that was prepared as described previously in Section 3.1.

**Figure 3:**
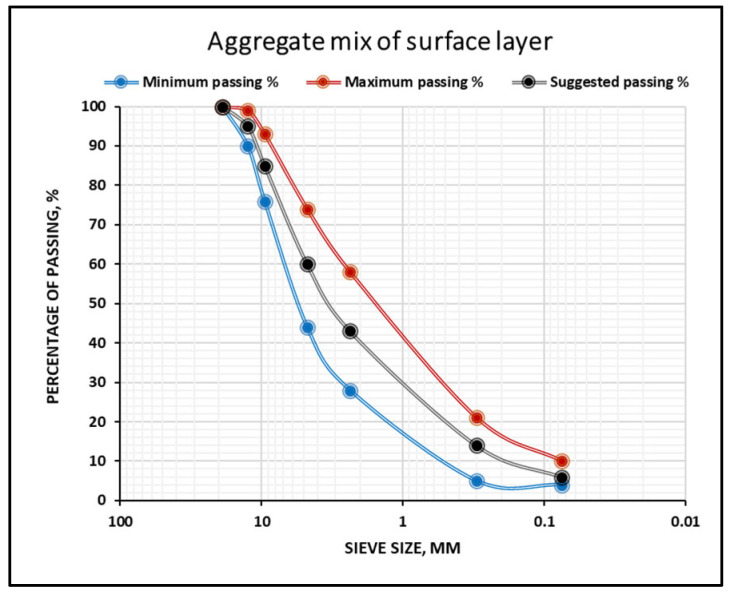
Gradation of aggregate mix for surface layer.

In this research work, both mixtures were designed based on the aggregate gradation of the surface layer as assigned by the SORB/R9 [35] and illustrated in Figure 3. Different sizes of natural coarse and fine aggregates, in addition to 6% limestone dust, were selected from local quarries, then sieved and combined. In both mixtures, the bitumen content (i.e., bitumen grade 40/50 and rubberized asphalt) was added in a certain amount and varied as 4%, 4.5%, 5%, 5.5%, and 6% to find out the optimum content.

For mixing purposes, aggregates were dried, heated, and mixed with heated binder at 160 °C, which is the designated temperature in the mechanical mixer to ensure that all aggregates were well-coated by asphalt and obtained a homogeneous mixture. Usually, three samples of mixture for each certain binder content were performed and placed in cylindrical molds (7.62 cm height and 10.16 cm diameter) for compacting in the Marshall machine at 135 °C. Then, the compacted samples of 1200 g weight were produced by subjecting 75 blows on the upper and bottom bases of molds using a hammer falling from a height of 46 cm. Finally, the compacted samples were prepared for the Marshall and rutting tests.

### 3.3. Statistical Analysis

For the experimental results to be more reliable by showing repeatability, every sample was tested in triplicate (*n* = 3), as per ASTM D6926. The result’s variability will be presented by the standard deviation (SD) and the coefficient of variation (CV). The results of the prepared mixtures will be discussed in the next section.

## 4. Results and Discussion

### 4.1. Resistance to Plastic Flow (Marshall Tests)

After preparing asphalt concrete samples for both controlled and modified mixtures as aforementioned in Section 3.2, the compacted samples were then tested in the Marshall apparatus to find out the optimum asphalt content (OAC) following the ASTM D6927-22 method. The test was conducted at a standard temperature of 60 °C and a loading rate of 2 inches/min. Mostly, the maximum load (Marshall stability) was recorded when the load was raised gradually to reach a peak. Then, when it was just beginning to fall, the loading was stopped. Also, the Marshall flow can be recorded at the same time the peak is obtained.

**Figure 4:**
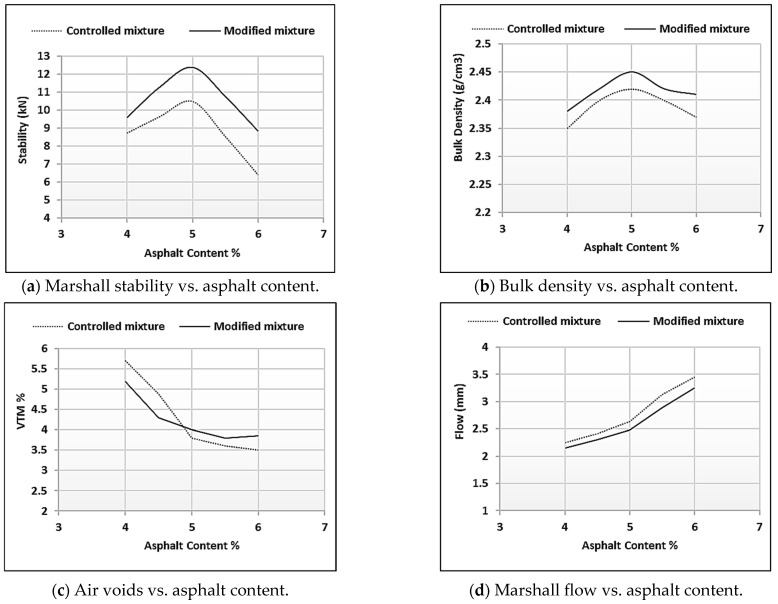
Results of the Marshall test for the reference and modified mixtures.

The optimum content of binder was investigated by achieving the maximum values of stability and density after adding specific amounts of asphalt. It is worth mentioning that the OAC can be obtained by taking the average of asphalt contents that meet the maximum density and maximum stability, and a certain percentage of air voids (usually 4%) in the total mixture. As shown in Figure 4, the OAC for the controlled mixture was 4.93%, while the OAC for the sustainable (modified) mixture was reported at 5.02%. This could be an advantage, as it has not changed much in terms of bitumen performance, considering economic aspects.

Table 6 presents the final results of both the controlled and modified mixtures, which showed good agreement with the requirements of SORB/R9 [35]. Marshall stability must be more than 8 kN according to SORB/R9 [35] and it increased by 18% after replacing asphalt grade 40/60 with rubberized asphalt in the mixture. Also, the Marshall flow was accepted within the required range between 2 and 4 mm, as stated in the Iraqi specifications [35], and fell around 5% compared to the controlled mixture. The percentage of air voids showed a slight difference; however, voids in mineral aggregates and those filled with asphalt in the modified mixture increased by 9.5% and 6%, respectively, compared to the controlled AC. To conclude, using rubberized asphalt might increase the stiffness of the mixture as well as reduce the void percentage in the total mixture.

**Figure 5:**
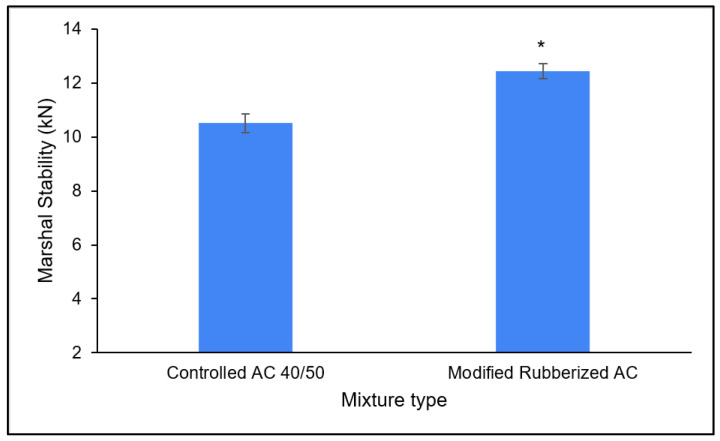
Marshall stability of controlled and modified asphalt mixtures (error bars represent the standard deviation * *p* < 0.05).

In Table 7, the statistical significance of the improvements in Marshall properties for the modified mixtures was assessed using a Student’s *t*-test at a 95% confidence level (*p* < 0.05). The Marshall stability of the rubberized mixture (12.45 kN) has shown a statistically significant improvement of 18.3% in comparison to the value of the control mixture (10.52 kN), as shown in Figure 5. The coefficient of variation was observed to be less than 3% (CV < 3%) across all tested samples. This confirms the high repeatability of values over mixing and testing procedures.

### 4.2. Performance (Rutting) Test

**Figure 6:**
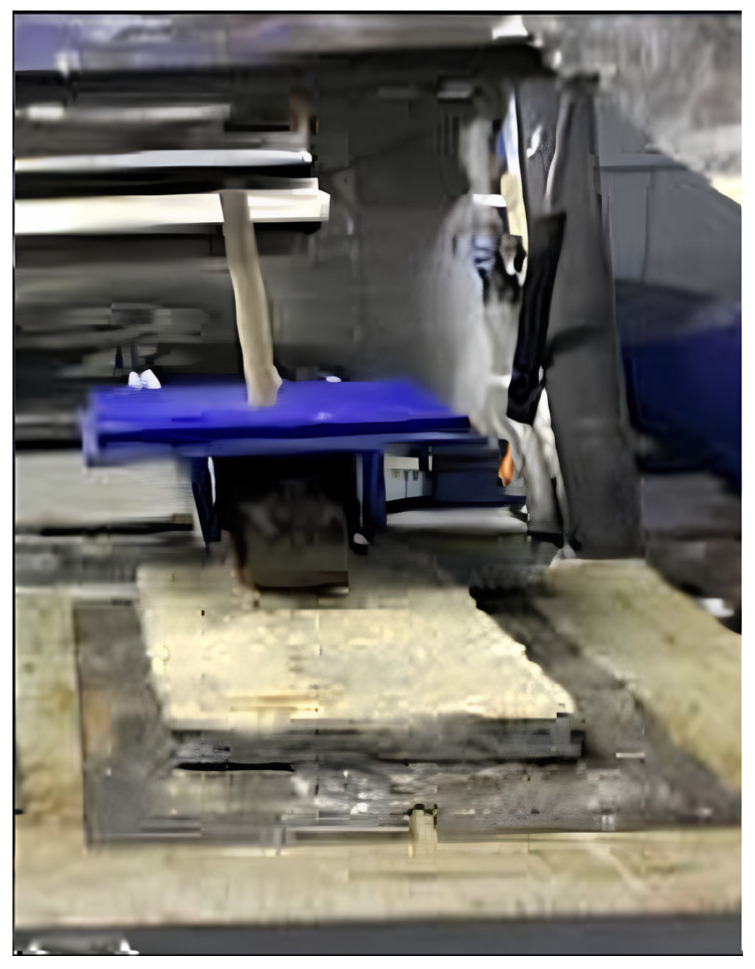
Examination of AC slabs in wheel track device.

This test was conducted to measure the resistance of the asphaltic surface to applied repeated loads. This test mimics the formation of rutting caused by axle loads as it occurs in the real world. Rutting depth can be estimated with the aid of the Hamburg wheel track under specific temperature conditions [30]. Therefore, compacted AC slabs of 180 mm × 300 mm and 50 mm thickness (i.e., controlled mixture in addition to modified mixture containing rubberized asphalt) were prepared for the test. In the next steps, the compacted slabs were placed in an oven for 4 h. Then, a steel wheel was rolled on the surface of compacted AC slabs with a velocity of 40 cycles/min and loads of 705 ± 4.5 N, as shown in Figure 6. The test was conducted at temperatures of 40 °C and 60 °C. As the rolling wheel applies compressive loads on the surface of the pavement model, the rutting deformation will occur after a specific number of repetitions. The wheel load runs for 20,000 passes (10,000 cycles) before achieving 20 mm rutting deformation.

**Figure 7:**
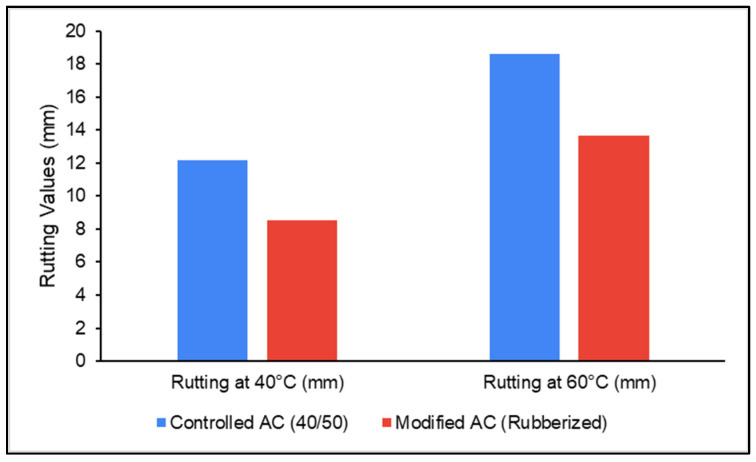
Comparison of rutting depth at temperatures of 40 °C and 60 °C.

Figure 7 presents the results of rutting behavior in reference and modified mixtures. It can be indicated that the prepared samples pass the requirement of SORB/R9 [35] and the rutting depths were recorded below 20 mm. Using rubberized asphalt in the pavement surface mixture provided positive results. About 30% reduction in rutting depth was recorded from 12.17 mm in the controlled mixture to 8.52 mm in the modified AC mixture at 40 °C. When the temperature increased to 60 °C, the recorded rutting depth decreased by 26% after replacing asphalt grade 40/50 with rubberized asphalt in the mixture.

This confirms that adding a specific amount of CR into asphalt might improve the mechanical properties of asphalt concrete, such as sensitivity to extreme temperature limits, and increase the rigidity of the mixture [37,38,39,40]. The results of this test are consistent with the Marshall quotient, which helps maintain the relationship between the tests.

## 5. Conclusions and Recommendations

In the current study, two main roads were investigated in Samawah city to assess the job mix design of the surface pavement mixture. Due to the absence of mixing management and control, in addition to heavy traffic loads, the road surface will start deteriorating, and distress will appear. Therefore, two types of asphalt were utilized in the preparation of asphalt mixtures in the current study. Asphalt type 40/50 grade was used in the preparation of the reference mixture, while CR particles were applied to modify asphalt type 60/70 with a specific content (5–20%) via the wet mixing method. Then, the rubberized asphalt was utilized to produce a modified mixture. In both mixtures, natural mixed aggregates in addition to limestone filler were used. Economically, using recycled rubber obtained from waste tires in producing modified asphalt is cheap in Iraq, in addition to its positive impact in reducing environmental pollution from waste polymers. The positive impact of using CR to modify 60/70 asphalt was reported, and it achieved lower penetration values and an increase in Marshall stability as the CR amount increased to 20%. As a result, the mechanical properties of sustainable asphalt concrete were improved after using rubberized asphalt in the mixture under different prevailing conditions. In comparison to the controlled mixture, the results of the sustainable mixture showed reduction trends in the rutting depths as 30% and 26% at 40 °C and 60 °C, respectively.

Furthermore, several recommendations can be highlighted for future studies, such as using different filler types and contents, for example, silica fume, fly ash, and glass powder, in sustainable asphalt concrete mixtures containing other types of filler for mixing with concrete aggregates from building waste. Resistance to plastic flow, moisture damage, and creep are seen, and fatigue tests can be conducted on these sustainable mixtures. Such approaches support circular economy principles and reduce environmental impacts, as highlighted in recent sustainability-focused transport material studies [41].

Also, it is necessary to follow the requirements of SORB/R9 [35] to achieve better results. Finally, quality control of mixed materials, lab tests, and designed layer thickness must be checked periodically to avoid surface deformation in the near future under prevailing weather conditions by performing Life-Cycle Cost Analysis (LCCA) to more thoroughly examine the various aspects of stability.

## Figures and Tables

**Table 1:** Laboratory tests of bitumen used in the current study.

Laboratory Tests	Units	Bitumen Grade 40/50	Bitumen Grade 60/70	Standard Test
Penetration: 100 g at 25 °C & 5 s	1/10 mm	45	64	ASTM D5
Ductility at 25 °C & 5 cm/min	cm	130	153	ASTM D113
Viscosity at 135 °C	C.s	520	405	ASTM D4402
Viscosity at 165 °C	C.s	253	93	ASTM D4402
Softening point (ring & ball)	°C	53	49	ASTM D36
Specific gravity at 25 °C	g/cm^3^	1.04	1.02	ASTM D70
Flash point (Cleveland Open Cup)	°C	250	233	ASTM D92

**Table 2:** Physical properties of natural fine aggregates.

Property	Units	Results	Standard Test
Bulk specific gravity	-	2.62	ASTM C127-88
Bulk SSD specific gravity	-	2.64	ASTM C127-88
Apparent specific gravity	-	2.68	ASTM C127-88
Absorption	%	1.2	ASTM C127-88
Air voids	%	45.5	ASTM C1252-23

**Table 3:** Physical properties of natural coarse aggregates.

Property	Units	Results	Standard Test
Bulk specific gravity	-	2.62	ASTM C127-88
Bulk SSD specific gravity	-	2.65	ASTM C127-88
Apparent specific gravity	-	2.69	ASTM C127-88
Absorption	%	1%	ASTM C127-88
Fractured particles	%	93% (Min 90%)	ASTM D5821-3
Resistance to degradation of mineral aggregate using LA abrasion machine	%	22% (Max 30%)	ASTM C131

**Table 4:** Physical properties of limestone dust.

Property	Units	Results	Standard Test
Passing sieve No. 200	%	100	ASTM C117
Plasticity index (PI)	%	0 (Max 4%)	SORB/R9
Apparent specific gravity	-	2.87	-
Specific surface area	m^2^/kg	392	-

**Table 5:** Modification of bitumen 60/70 by adding CR contents.

Laboratory Tests	Units	Bitumen Grade 60/70	CR Content %						ASTM Standard Tests
			5	10	12.5	15	17.5	20	
Penetration 100 g, 25 °C, 5 s	1/10 mm	64	52	51	50	48	46	43	D5
Ductility at 25 °C, 5 cm/min	cm	153	146	137	125	117	108	96	D113
Softening point (ring & ball)	°C	49	51	51	52	53	54	56	D36
Specific gravity at 25 °C	-	1.02	1.02	1.02	1.02	1.02	1.02	1.02	D70
Flash point (Cleveland Open Cup)	°C	233	239	243	248	251	253	255	D92

**Table 6:** Summary of prepared asphalt mixtures according to SORB/R9 requirements.

Marshall Properties	Controlled AC Containing Asphalt Grade 40/50	Modified AC Containing Rubberized Asphalt	SORB/R9 Specification for Surface Layer [35]	Remarks
Optimum asphalt content (OAC), %	4.93	5.02	4.0–6.0	Accepted
Marshall stability, kN	10.52	12.45	Min 8.0	Accepted
Marshall flow, mm	2.64	2.50	2.0–4.0	Accepted
Marshall quotient, kN/mm	3.98	4.98	Min 2.0	Accepted
Voids in total mix (VTM), %	3.93	4.00	3.0–5.0	Accepted
Voids in mineral aggregate (VMA), %	15.18	19.40	Min 14	Accepted
Voids filled with asphalt (VFA), %	72.61	71.25	65–85	Accepted

**Table 7:** Statistical values of mixture properties based on optimum asphalt content (OAC).

Mixture Property	Controlled AC (Average)	Modified AC (Average)	Standard Deviation (σ)	Coeff. of Variation (CV)
Marshall Stability, kN	10.52	12.45	±0.35	0.028
Marshall Flow, mm	2.64	2.5	±0.12	0.048

## Data Availability

The data presented in this study are available in the article.

## Abbreviations

The following abbreviations are used in this manuscript:

AASHTO	American Association of State Highway and Transportation Officials
ASTM	American Society for Testing and Materials
AC	Asphalt concrete
CR	Crumb rubber
OAC	Optimum asphalt content
SORB/R9	Iraqi General Specifications for Roads and Bridges
